# A comprehensive and accessible model for co-segregation analysis in *BRCA1*, *BRCA2*, and *PALB2* variant classification

**DOI:** 10.1007/s00439-025-02816-z

**Published:** 2026-03-26

**Authors:** Setareh Moghadasi, Ramin Monajemi, Merel E. Braspenning, Maaike P. G. Vreeswijk, Mar Rodríguez-Girondo

**Affiliations:** 1 Department of Clinical Genetics, Leiden University Medical Center, Leiden, The Netherlands; 2 Department of Biomedical Data Sciences, Leiden University Medical Center, Leiden, The Netherlands; 3 Department of Human Genetics, Leiden University Medical Center, Leiden, The Netherlands

## Abstract

Variants of uncertain significance (VUS) are genetic variations with unclear clinical implications, often complicating clinical management in genetic testing. The analysis of co-segregation of the variant with the disease in families has been shown to be a powerful tool for the classification of these variants. We present CAL-Leiden (Co-segregation Analysis via Likelihood ratio analysis-Leiden), a comprehensive co-segregation model facilitating the classification of variants in *BRCA1*, *BRCA2* and *PALB2* genes, which can be used as an important component of the ACMG/AMP (the American College of Medical Genetics and Genomics/ the Association for Molecular Pathology) classification guideline. CAL-Leiden includes an expanded range of cancer types, including pancreatic cancer, in addition to breast and ovarian cancer. The model operates on a multiple-cancers-per-individual framework, so that likelihood contributions account for all relevant cancers observed in a person, including contralateral breast cancer. The model integrates population incidence rates from the Netherlands, the United Kingdom and United States, along with penetrance data from the latest literature. A web-based platform has been developed, making the model accessible and practical for use in diagnostic labs: https://bioexp.net/cosegregation/. We demonstrate the functionality of the tool with multiple pedigrees and compare its performance with alternative approaches. The features in CAL-Leiden collectively contribute to a more comprehensive and accurate assessment of variant pathogenicity, helping clinical laboratory specialists and researchers in classification of the variants of uncertain significance.

**Supplementary Information:**

The online version contains supplementary material available at 10.1007/s00439-025-02816-z.

## Introduction

The broad application of sequencing technologies in DNA diagnostic laboratories and the use of extensive cancer related gene panels have resulted in the identification of a growing number of sequence variants in major cancer-predisposition genes such as *BRCA1*, *BRCA2* and *PALB2*, of which the clinical importance remains uncertain, referred to as Variants of Uncertain Significance (VUS). A variety of approaches have been used to assess the clinical relevance of these VUS, among which co-segregation analysis.

Co-segregation analysis studies the inheritance of a genetic variant within a family, particularly its presence in affected or unaffected family members, to determine the likelihood of its pathogenicity or association with a specific disease. Different statistical approaches have been described to determine the likelihood of pathogenicity using co-segregation data which can then be integrated together with supplementary evidence for the application in ACMG/AMP-based classification in which all the available data against or in favor of pathogenicity are combined (Tavtigian et al. 2018).

The major contribution in the early 2000s was the introduction of the full pedigree likelihood model (Thompson et al. 2003). This method proposed the concept of assessing the probability that observed genetic patterns within family pedigrees align with a predefined gene-disease penetrance model. The method employed incidence rates to depict the penetrance for affected individuals. However, these might not be entirely suitable for age-dependent phenotypes such as cancer age of onset. Moreover, within the full pedigree approach, the penetrance was assumed to remain constant across 10-year time intervals (liability classes during which penetrance is constant), which may not accurately capture biological reality. It also required specialized linkage software, limiting accessibility.

In 2009, Mohammadi et al. (Mohammadi et al. 2009) introduced a novel survival-based pedigree likelihood model that considers an individual's entire lifespan: affected individuals contribute via a density function, and unaffected individuals via the survival function at last follow-up. Additionally, The model incorporates multiple cancers per individual, including breast cancer (BC), ovarian cancer (OC), and contralateral breast cancer (CBC), allowing likelihood contributions to reflect all relevant diagnoses and absence of disease. A web-based tool was also provided to facilitate pedigree analysis without specialized software.

Recently, two papers were published using a similar survival approach. Rañola et al. (2018) presented an adaptation of the Mohammadi model, integrating the latest age-dependent BC penetrance data for *BRCA1* and *BRCA2* carriers and other genes such as *ATM*, *CHEK2*, *MEN1*, *MLH1*, *MSH2*, *MSH6*, and *PMS2* were included. However, this adaptation simplified the original Mohammadi penetrance model; focusing solely on the first cancer. More recently, Belman et al. (Belman et al. 2020) revised the original full pedigree likelihood model (Thompson et al. 2003) by incorporating a survival formulation in the likelihood specification resulting in a similar statistical formulation to the Mohammadi model (Mohammadi et al. 2009).

This study presents an updated version of the Mohammadi model, with several enhancements: inclusion of *PALB2* alongside *BRCA1* and *BRCA2*, incorporation of pancreatic cancer, and updated penetrance data from the Netherlands, United Kingdom (UK) and United States (US). The model continues to account for multiple cancers per individual. A new web application (https://bioexp.net/cosegregation/) has been launched. We evaluated the performance of the tool with multiple pedigrees and compared the results with alternative approaches by Rañola et al. (Rañola et al. 2018) and Belman et al. (Belman et al. 2020) as were published in Belman. These improvements contribute to a more reliable assessment of variant pathogenicity and help the clinical laboratory specialists in the classification of these variants.

## Subjects and methods

### Mohammadi et al. (2009) revised and extended

The likelihood ratio (LR) for variant pathogenicity is defined as the probability of observing the family genotypes given the family disease phenotypes under the pathogenic hypothesis, divided by the probability of observing the same genotypes under the benign hypothesis:

1 $$LR=\frac{{P}_{d}({G}_{o}|{Ph}_{f},{G}_{p}=1)}{{P}_{n}({G}_{o}|{Ph}_{f},{G}_{p}=1)}$$

where *Ph*_*f*_ denotes the observed phenotypes in family *f*, *G*_*O*_ the genotypes of the VUS in family *f, *and* G*_*p*_ = *1* indicates that the proband is a carrier. For benign variants, the LR is close to 1; for pathogenic variants, LR > 1. Higher LR values indicate stronger evidence of pathogenicity. Detailed derivations are provided in Mohammadi et al. (2009) and Appendix A of the Supplementary Material.

The key components in the *LR* are the individual likelihood contributions under pathogenic and benign assumptions based on phenotypical information. For each individual, phenotypical information includes the age at cancer onset for gene-associated cancers, or the age at the last examination for unaffected individuals. In the original Mohammadi model, three cancers were considered: BC, OC and CBC. For female *i*, the data is given by (*t*_*bci*_,*t*_*ovi*_,*t*_*cbci*_,*d*_*bci*_,*d*_*ovi*_,*d*_*cbci*_*)*, where *d*_*bci*_ = 1 if female *i* is affected by BC and 0 otherwise and *t*_*bci*_ is age of BC diagnosis or last examination if unaffected. Analogously, *t*_*ovi*_ is the age at OC diagnosis or last examination if unaffected and *d*_*ovi*_ = 1 if female *i* is affected by OC and 0 otherwise. Finally, *t*_*cbci*_, where *t*_*cbci*_>*t*_*bci*,_ is the age at CBC diagnosis and *d*_*cbci*_ = 1 if affected by CBC^.^ If unaffected at the end of follow-up, *t*_*bci*_ = *t*_*ovi*_ = *t*_*cbci*_ are the age at the last examination and *d*_*bci*_ = *d*_*ovi*_ = *d*_*cbc*i_= 0.

Assuming independence of BC and OC diagnosis, and based on standard likelihood theory for survival data, the likelihood contribution of female *i* is given by:

2 $$P({Ph}_{i})=\left\{\begin{array}{c}{f}_{bc}\left({t}_{bci}\right){f}_{ov}\left({t}_{ovi}\right) \mathrm{if} \;{d}_{bci}=1 \;\mathrm{and}\; {d}_{ovi}=1\\ {f}_{bc}\left({t}_{bci}\right){S}_{ov}\left({t}_{ovi}\right) \mathrm{if} \;{d}_{bci}=1 \;\mathrm{and}\; {d}_{ovi}=0\\ {S}_{bc}\left({t}_{bci}\right){S}_{ov}\left({t}_{ovi}\right) \mathrm{if} \;{d}_{bci}=0 \;\mathrm{and}\; {d}_{ovi}=0\\ \end{array}\right.$$

where *S*_*bc*_(*t*_*bci*_*)* = 1-*F(t*_*bci*_*)* and *S(t*_*ovi*_*)* = 1-*F(t*_*ovi*_*)* are the probabilities of being free of BC and OC by age *t*_*bci*_ and *t*_*ovi.*_. *F*_*bc*_*(t*_*bci*_*)* and *F*_*ov*_*(t*_*ovi*_*)* are the penetrances, i.e. the cumulative risks of having BC and OC before ages *t*_*bci*_* and t*_*ovi*_, and *f*_*bc*_*(t*_*bci*_*)* and *f*_*ov*_*(t*_*ovi*_*)* are their corresponding derivatives, the density functions. Each density can be rewritten as *f(t*_*i*_*)* = *h(t*_*i*_*)S(t*_*i*_*)*, highlighting that the likelihood for an affected individual at age* t*_*i*_ combines the probability of remaining cancer-free until age *t*_*i*_ with the instantaneous probability of developing cancer at age *t*_*i*_*.* Penetrances *F(t)* were smoothed from published data using normal distributions (Jonker et al. 2003).

For CBC, the likelihood term *f*_*bc*_*(t*_*bci*_*)* in expression (2) was replaced by $$\raisebox{1ex}{${f}_{bc}({t}_{bci}){f}_{bc}({t}_{cbci})$}\!\left/ \!\raisebox{-1ex}{$4\sqrt{{S}_{bc}({t}_{bci}){S}_{bc}({t}_{cbci})}$}\right.$$, assuming independence of cancers in each breast. Conditional probabilities of CBC were unavailable at the time, so BC penetrance was treated as the minimum of these two independent processes. Details are in Supplementary Appendix B.

The independence assumption in expression (2) implies that after one gene-related cancer, the risk of a second cancer remains unchanged. In reality, surveillance or treatment may modify this risk. A pragmatic solution to avoid this independence assumption is to modify the dataset so that the follow-up for each individual ends at the age of the first cancer diagnosis. The Mohammadi model an then be applied without using the multiple cancer occurrences.

Expression (2) aligns with the penetrance formulation proposed by Belman et al. (2020), which uses the same phenotypic likelihood formulation but enforces stopping follow-up after the first cancer diagnosis. The approach by Rañola et al. (2018) is less comprehensive: only the first occurring BC or OC is used to define the affected status. However, the penetrance for unaffected individuals solely considers BC. Neither Belman et al. (2020), nor Rañola et al. (2018) models CBC. Derivations and a detailed model comparison among the three methods is provided in the Appendix C of the Supplementary Material.

### Extension of the Mohammadi model: more genes, cancer types, and updated penetrances

The Mohammadi model (Mohammadi et al. 2009) was extended in three main directions: (i) inclusion of the *PALB2* gene, (ii) incorporation of pancreatic cancer, and (iii) updating of penetrances based on the latest literature.

The integration of multiple cancers follows the same principle illustrated for BC and OC in expression (2). Age-specific cancer incidence rates and corresponding cumulative risks used in expression (2) were updated accordingly. Five-year age-specific cancer incidence rates for carriers of pathogenic variants in *BRCA1*, *BRCA2*, and *PALB2* were obtained by multiplying the underlying population incidence rates by the most recently published age-specific relative risks (Antoniou et al. 2008; BCLC 1999; Kuchenbaecker et al. 2017; Mocci et al. 2013; Thompson and Easton 2002; Yang et al. 2020). A complete list of cancers types per gene, with the updated parameters and references, can be found in Tables 1 and 2, and in Figure 1 in the Supplementary Material..

**Table 1:** Likelihood ratios (LRs) for varying affection status and genotype of individual “?” in the pedigree from Fig. 1

	Rañola et al. (2018)	Mohammadi et al. (2009)	CAL-Leiden	COOL (Belman et al. 2020)
	As published by Belman et al.	In-house program	https://bioexp.net/cosegregation/	fengbj-laboratory.org
Affection status and genotype of person “?”				
Affected, Variant present	36.9	15.13	6.58	3.85
Unaffected, Variant present	9.06	6.12	2.85	3.85
Affected, Variant absent	17.3	24.35	15.19	19.7
Unaffected, Variant absent	42.5	30.88	16.86	19.7
Affected, Untested	27.1	19.74	10.88	11.8

LRs are reported for the Rañola et al. approach (as in Belman et al. 2020), Mohammadi’s co-segregation likelihood model (our in-house model), CAL-Leiden (this paper), and COOL (as reported in Belman et al. 2020). Although COOL reports a Bayes Factor (BF), these values are equivalent here, both compare the probability of the observed data under pathogenic versus benign hypotheses, so the term LR is used throughout. CAL-Leiden and COOL analyses were run using UK population data; COOL used a default allele frequency of 0.0001

Three reference populations were considered: the Netherlands, the United Kingdom, and the United States.Population data were obtained from national cancer registries for the Netherlands (IKNL, 2017),the United Kingdom (Cancer Research UK, 2015–2017), and the United States (SEER, 2018–2022). Penetrance incidence data for other populations can be incorporated into the model upon request. For more information, please contact the corresponding author.

Following the original Mohammadi approach, we obtained smoothed versions of the penetrance based on a truncated normal distribution with parameters *r* (lifetime cancer risk), *μ* (mean age of cancer onset), and *σ* ( standard deviation of the age of cancer onset).

For each cancer type and gene, the optimal values of *r*, *μ σ* were obtained minimizing sum of squared differences between the observed incidence rates and the theoretical values derived from the hazard function of a normal distribution scaled by a cumulative risk factor (r). Let *t*_*i*_ ​ denote the age and *y*_*i*_ the observed incidence rate for age group 𝑖; the data were fitted using the following objective function:

3 $$Sum \;of \;Squares=\sum {y}_{i}-\frac{r{f}_{norm}({t}_{i},\mu ,\sigma )}{1-r{F}_{norm}({t}_{i},\mu ,\sigma )}$$

MetaMeta

where ${f}_{norm}$, is the normal density function and ${F}_{norm}$ is the corresponding cumulative distribution function.

For CBC, we introduced a new formulation that does not assume independence between primary and contralateral breast cancers. Using conditional relative risk data for *BRCA1* and *BRCA2* carriers (Kuchenbaecker et al. 2017) and for the general population (Peto and Mack 2000), the joint density was modeled as:

4 $${f}_{bc}\left({t}_{bci},{t}_{cbci}\right)={f}_{bc}\left({t}_{bci}\right){f}_{cbc}\left({t}_{cbci}|{t}_{cbi}\right)={f}_{bc}\left({t}_{bci}\right){f}_{cbc}({t}_{cbci}-{t}_{bci})$$

The conditional term ${f}_{cbc}({t}_{cbci}-{t}_{bci})$ was smoothed using an exponential (constant-hazard) model, as CBC incidence rates showed no clear age-varying trend. Further details on the modeling of CBC can be found in the Appendix B of the Supplementary Material.

### A new web application

We developed a user-friendly web application with pedigree visualization, intuitive data entry, and automated report generation (https://bioexp.net/cosegregation/). The pedigree input format is fully compatible with CanRisk (https://www.canrisk.org/), widely used for personalized risk assessments of BC and OC and recognized as a medical device in clinical practice. Since CanRisk-formatted pedigree files are often available, using the same structure facilitates data reuse and minimizes preprocessing.

CAL-Leiden allows flexible handling of multiple cancers per individual. Via a drop-down menu, users can include all gene-related cancers, account for contralateral breast cancer (CBC), or censor follow-up at the first cancer diagnosis without modifying the input data.

As in Mohammadi et al. (2009), CAL-Leiden relies on a computational algorithm that enumerates all genotypic configurations of a rare variant. The algorithm has been optimized for improved speed and reduced memory usage compared to the original implementation. However, in large or complex pedigrees, calculation of the likelihood ratio (LR) may still fail due to algorithmic limitations. In such cases, it is advisable to trim branches containing only unaffected and/or untested relatives, particularly individuals under 20 years of age, consistent with Mohammadi et al. (2009).

Privacy and data security were prioritized during development. Uploaded pedigrees are not retained on the server. Users can generate a PDF report of their analyses for local archiving. The source code is available upon request; please contact the corresponding author for details.

## Results

**Fig.1:**
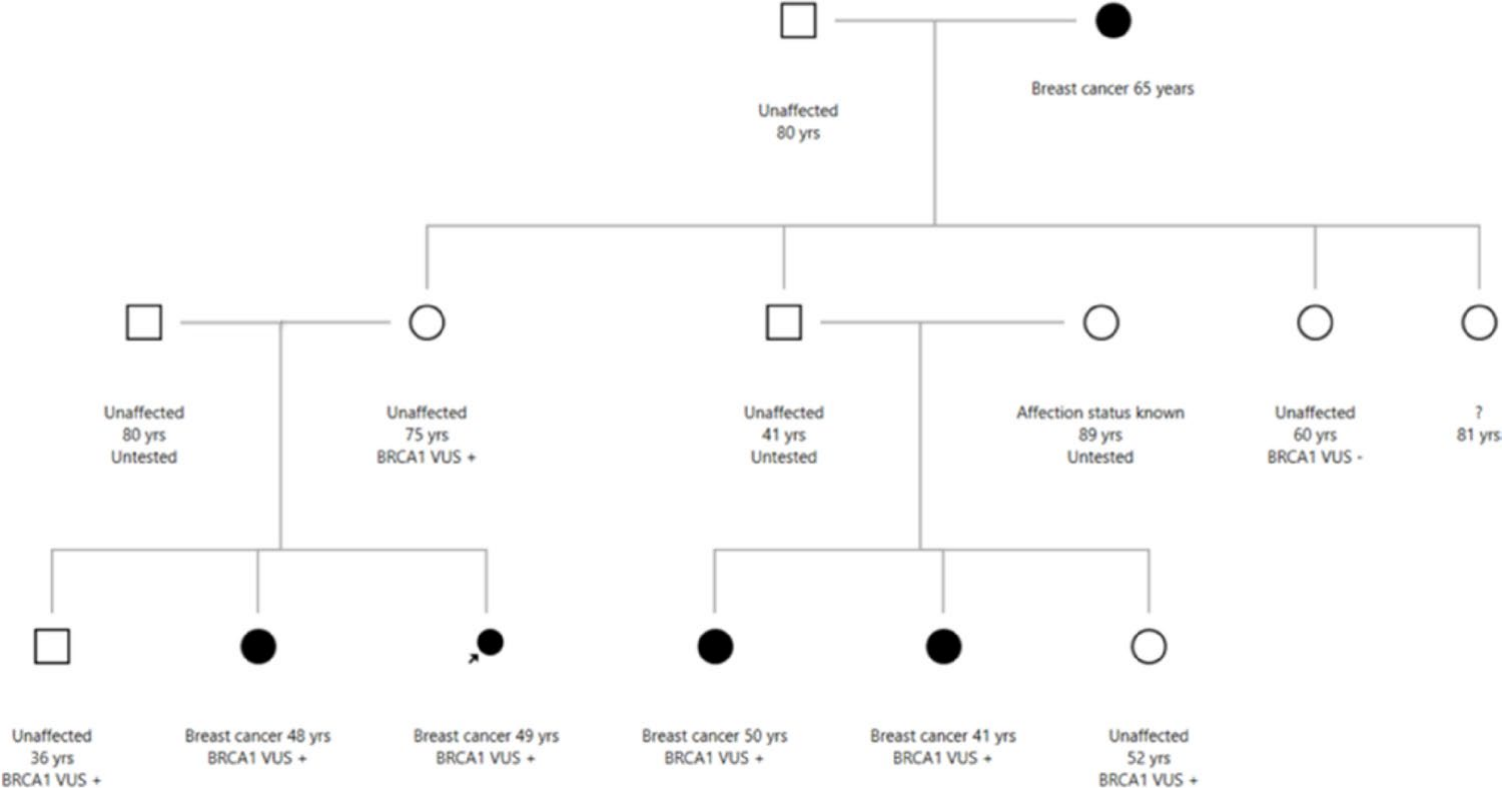
Pedigree based on Fig. 1 in Belman et al. (2020). In this pedigree Proband is indicated with an arrow. Black filled: affected with breast cancer. Age of onset for affected and age of the last follow-up for unaffected individuals is indicated. Genotype is indicated as “BRCA1 VUS + for carrier of the VUS BRCA1 VUS- for not carriers of the VUS in the BRCA1. For person named “?” affected status and genotype is to be determined.

**Fig. 2:**
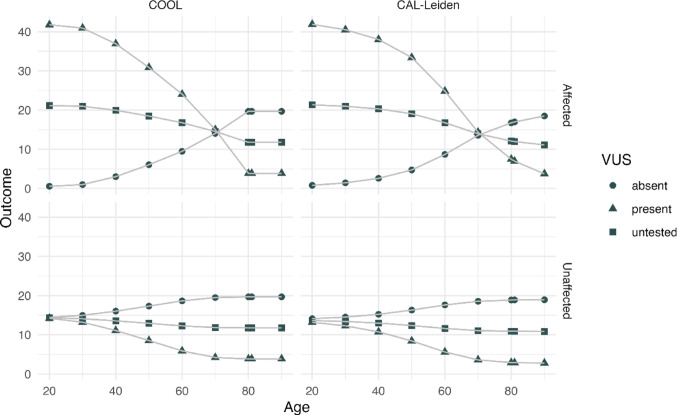
LRs by COOL (Belman et al. 2020) and CAL-Leiden for person ? in Fig. 1, at different ages given different affected status and genotype. Although COOL reports a Bayes Factor (BF), these values are equivalent here, both compare the probability of the observed data under pathogenic versus benign hypotheses, so the term LR is used throughout. CAL-Leiden and COOL analyses were run using UK population data; COOL used a default allele frequency of 0.0001.

After implementing the updates described in the methods section, we conducted a side-by-side comparison between the output of CAL-Leiden, Mohammadi et al. (2009), COOL (version 2) (Belman et al. 2020), and the Rañola et al. (2018) approach as implemented in the R package Coseg, respectively. For clarity and ease of comparison, our primary analysis was based on the same pedigree described in Belman et al. (2020). We revisited the impact of different affected and carrier status of the 81-year-old female family member (Fig. 1, Table 1) and extended the analysis by varying her age from 20 to 85 years (Fig. 2).

Table 1 is based on Belman’s Table S3, in which they compared the performance of their model (COOL) with the Rañola et al. model (Rañola et al. 2018), finding large discrepancies between the two. When we run the original Mohammadi model (Mohammadi et al. 2009), along with the updated model (CAL-Leiden) and compare them to COOL (Belman et al. 2020) we see comparable results between COOL and CAL-Leiden. This similarity indicates that the primary differences with the original Mohammadi model (Mohammadi et al. 2009) are due to outdated penetrance data with higher lifetime risks for carriers, rather than a fundamentally different penetrance model. The original Mohammadi penetrances are provided in Supplementary Table 3, allowing direct comparison with the updated parameters in Supplementary Table 1 and highlighting the sources of the differences observed. An overview of key characteristics across the different models is given in Supplementary Table 4.

When considering the individual as affected or unaffected, along with the presence or absence of the variant or unknown variant status across a grid of ages from 20 to 85, both COOL and CAL-Leiden follow a comparable curve (Fig. 2). The slight differences between CAL-Leiden and COOL can be attributed to variations in the underlying population data and differences in the age-specific incidence rates modeling. CAL-Leiden uses a normal-based smoothing approach, while COOL directly employs raw, stepwise constant age-specific age rates. This difference in the modeling of age-specific rates explains the slight divergence between the two models beyond the age of 80. In COOL, when the affected status is altered beyond the age of 80, the LR remains numerically equal. This is because COOL enforces a fixed relative risk of cancer of 1 beyond 80 years of age. In contrast, CAL-Leiden, without this constraint due to its smoothing approach, results in closely aligned but decreasing LRs beyond 80.

While the differences in modeling of age-specific incidence rates generally have a small impact on BC, they might significantly affect OC. This is due to important fluctuations in the raw data caused by the lack of events at young ages. Supplementary material Fig. 2 illustrates the effect of smooth versus stepwise penetrance for all three genes in using a simple pedigree in which OC is present. In the case of *PALB2*, the LR in COOL (Belman et al. 2020) exhibits significant fluctuations, indicating that the likelihood of pathogenicity is lower when OC occurs between ages 40 and 50 (3.662) compared to diagnosis between ages 50 and 60 (4.489). Furthermore, OC between ages 60 and 70 shows an even lower likelihood (2.266), while diagnosis between ages 70 and 80 exhibits a higher likelihood (2.934).

Additionally, we studied the performance of CAL-Leiden in three pedigrees with varying sizes and structures for *BRCA1*, *BRCA2* and *PALB2* (supplementary material Fig. 3). The results demonstrate that the CAL-Leiden model can handle different sizes of pedigrees. Furthermore, the results obtained from CAL-Leiden and COOL (Belman et al. 2020) exhibit again a high degree of comparability in these pedigrees.

Finally, in supplementary material Fig. 4, we show how different and multiple within-person cancer types impact the LR in CAL-Leiden within a fixed pedigree configuration for *BRCA1*, *BRCA2* and *PALB2* genes. These examples also show the expected differences when comparing models that consider all cancers per person versus those that censor follow-up after the first diagnosis: LRs are generally higher when all cancers are considered, although the magnitude of this increase depends on the specific combination of cancers.

## Discussion

We revised the Mohammadi co-segregation likelihood model (Mohammadi et al. 2009) by incorporating updated population incidences and relative risks, adding pancreatic cancer into the algorithm, including the analysis for *PALB2* and updating the modeling for CBC. Furthermore, we have developed a website to facilitate the assessment of co-segregation LR using the penetrance based on the cancer incidence for Dutch, UK, or US population. Regarding our website we have prioritized designing an interface that is user-friendly. Upon uploading the pedigree in a CanRisk table format, it is displayed on the screen for visual inspection and confirmation, eliminating the need for manual input. Population incidence for the UK, NL, and US are also available in dropdown menus, so users do not need to provide these incidences themselves. After applying the calculation button, the corresponding LR is displayed in the same interface and results can be downloaded as a PDF for personal records. To address patient privacy concerns, especially regarding cross-border data exchange, the model is available upon request. This allows users to process pedigrees within their own institute without uploading data to external servers.

Belman et al. (2020) implied that the discrepancies in results between COOL and Mohammadi et al. (2009) and Rañola et al. (2018) model stem from the use of a ‘cumulative risk model’. However, it should be noted that a model based on cumulative risk has never been described by Mohammadi et al. As explained above, both the Mohammadi model and the Rañola adaptation of it rely on a survival approach, similar to the approach used by COOL (Belman et al. 2020). As shown in the results section, CAL-Leiden and COOL (Belman et al. 2020) show overall comparable results. The observed slight differences can presumably be attributed to differences in population incidences, relative risks, and differences in the age-specific incidence rates modeling. In CAL-Leiden, the raw data, based on age intervals with constant incidence rates, is smoothed using the best-fitting normal approximation. This leads to smooth estimates of the age-specific age rates, reducing spurious fluctuations in the data caused by combining age-dependent relative risks and underlying population incidence rates of non-coinciding intervals. On the other hand, COOL (Belman et al. 2020) uses the original raw age-specific cancer incidence rates based on intervals. The practical impact on LR of this different approach is small.

In some cases, individuals within a pedigree may be diagnosed with multiple gene-related cancers, such as BC and OC, or CBC. Both the original model of Mohammadi et al. (2009) and CAL-Leiden account for CBC, but in contrast to the independence assumption applied in the original model, CAL-Leiden integrates newly published risk estimates for CBC that adjust for age and time since the first primary BC diagnosis (Kuchenbaecker et al. 2017).

For other cancer combinations, however, reliable published data on co-occurrence risks are still lacking. In light of this, CAL-Leiden offers users three options: to treat each cancer diagnosis as an independent event, to end follow-up after the first relevant cancer diagnosis, or to apply an hybrid approach where follow-up ends after the first diagnosis except for CBC, which is handled using the specific aforementioned risk model based on recent literature. These scenarios can be selected directly in the user interface without modifying the input data. For example, selecting the “All relevant cancers” considers all gene-related cancers diagnosis as independent processes, except CBC which is considered as explained above using the most up-to-date published risk data. One might prefer, however, to end the follow-up at the first cancer diagnosis. This alternative approach is also available in CAL-Leiden: “First diagnosis”, allows users to choose this option, without modifying the input data. This option mirrors the approach used in COOL (Belman et al. 2020) and aligns with the current guidelines of the Variant Curation Expert Panel (VCEP) (https://clinicalgenome.org/affiliation/50039). However, such an approach has the downside of discarding potentially relevant information with respect to the pathogenicity of the variant under investigation, leading to underestimation of the variant's pathogenicity likelihood. For example, when a VUS is identified in a woman with both BC and OC diagnosed at a young age, it is more likely that the variant is pathogenic compared to when she has had only BC. For those who wish to end follow-up after the first cancer diagnosis but want to include CBC after the first BC, the “First diagnosis + CBC” option is available.

This combination of flexibility, allowing users to explore different assumptions when specific co-occurrence data are lacking, together with the ability to incorporate new risk estimates as they become available, is a key strength of CAL-Leiden. As demonstrated for CBC, the model can readily integrate new data on cancer co-occurrence, and as more information becomes available for other cancer types, it will be incorporated.

Beyond the issue of multiple cancers, our work also highlights the broader need for methodological transparency in the field. Such transparency is critical to ensure comparability across studies and reproducibility in clinical variant interpretation. By offering both a detailed theoretical framework and practical examples, CAL-Leiden aims to support a deeper understanding of how different modeling strategies approach co-segregation analysis and encourage informed application of these tools. Recently, the web-based application ShinySeg (Carrizosa et al. 2024) was released to facilitate co-segregation analyses through an interactive interface. While it focuses on sensitivity analyses for penetrance assumptions, it does not specify the likelihood formulation or provide a formal model for variant classification. As such, it differs in scope and purpose from our approach.

The inclusion of prostate cancer in the co-segregation calculations is a subject of ongoing debate. Previous research has firmly linked pathogenic *BRCA2* variants to an increased risk of prostate cancer (BCLC 1999; Kote-Jarai et al. 2011). The European Randomized Study of Screening for Prostate Cancer (ERSPC) demonstrated reduced mortality with PSA-based screening (Kohestani et al. 2018; Page et al. 2019), leading to its implementation in several countries. As a result, many prostate cancers are now detected through PSA screening, including a substantial proportion of low-risk tumors that may not reflect the underlying genetic risk. The presence of these screen-detected cases can therefore introduce bias into co-segregation analyses. Ideally, we would exclude low-risk prostate tumors detected solely through PSA screening. However, we cannot ensure that users will consistently have access to the detailed clinical information needed to distinguish these cases when preparing the data file. For this reason, we have chosen not to include prostate cancer in the calculations.

While CAL-Leiden shows improved computational performance over the original Mohammadi et al. (3) implementation, its underlying enumeration algorithm can still limit calculations in large or complex pedigrees. To address this, we are exploring the use of alternative approaches, such as the Elston-Stewart algorithm (Elston 2005; Elston and Stewart 1971), which may improve scalability and allow for efficient analysis of more complex family structures in future versions.

Quantitative co-segregation analysis methods are well established for their use in interpretation of causality of *BRCA1*, *BRCA2* and *PALB2* variants in ACMG/AMP guidelines. The LRs can be converted to different PP1 (supporting strength for pathogenicity) and BS4 (lack of segregation in affected members of a family) strength categories, following the LR thresholds described in Tavtigian et al. (Tavtigian et al. 2018). If multiple independent families carry the same variant, their LRs can be multiplied. Differences in LR between different co-segregation models could potentially bear significance when the LR approaches the ACMG threshold for a specific strength criterion. However, it is worth noting that in many instances, the differences in LR are minimal, rendering this an often inconsequential issue. Given the substantial similarities between the tools, the choice of which model to employ may be influenced by user familiarity, specific (safety) rules and regulations, or other nuanced considerations.

Additionally, it is important to recognize that none of the existing models for VUS classification, relying on co-segregation data, currently incorporate measures of uncertainty into their outcomes. This issue becomes particularly significant when the LR is derived from a limited number of families. To address this challenge, it is important to enhance the confidence in the classification by combining the results from unrelated families carrying the same VUS. International collaborations like the ENIGMA consortium facilitate data aggregation, ultimately leading to a more reliable reclassification of VUS. This, in turn, holds utmost significance for patient care and treatment decisions.

## Supplementary Information

Below is the link to the electronic supplementary material.

Supplementary Material 1

## Data Availability

For pedigree outputs in the main manuscript or Supplementary Material, please contact the authors. The population-level data used in this study were obtained from publicly available national cancer registries. Data for the Netherlands were accessed from the Netherlands Comprehensive Cancer Organisation (IKNL) (https://iknl.nl).Data for the United Kingdom were obtained from Cancer Research UK (https://www.cancerresearchuk.org). Data for the United States were obtained from the Surveillance, Epidemiology, and End Results (SEER) program (https://seer.cancer.gov).

## Code availability

For the code used in the model, please contact the authors.
