# Correction: A comprehensive and accessible model for co-segregation analysis in *BRCA1*, *BRCA2*, and *PALB2* variant classification

**DOI:** 10.1007/s00439-026-02844-3

**Published:** 2026-06-10

**Authors:** Setareh Moghadasi, Ramin Monajemi, Merel E. Braspenning, Maaike P. G. Vreeswijk, Mar Rodríguez-Girondo

**Affiliations:** 1 Department of Clinical Genetics, Leiden University Medical Center, Leiden, The Netherlands; 2 Department of Biomedical Data Sciences, Leiden University Medical Center, Leiden, The Netherlands; 3 Department of Human Genetics, Leiden University Medical Center, Leiden, The Netherlands

**Correction: Human Genetics (2026) 145:34** 10.1007/s00439-025-02816-z

In this article, there is a typo error in the body text, now it has been corrected.

In the sentence beginning, “where…”, the word ‘and’ should be in normal letters.

In the sentence beginning, “Finally…”, the word ‘cbi’ should be subscript.

Formula (2) before ‘if’ space ‘and’ before and. if and ‘and’ are text. These are conditional formulas, so it has to be possible to read it, differentiate the words. Also, not italic for ‘if’ and ‘and’.

The original article has been corrected.

